# Asthmatic allergen inhalation sensitises carotid bodies to lysophosphatidic acid

**DOI:** 10.1186/s12974-021-02241-9

**Published:** 2021-08-31

**Authors:** Nicholas G. Jendzjowsky, Arijit Roy, Richard J. A. Wilson

**Affiliations:** 1 Department of Respiratory Medicine and Exercise Physiology, The Lundquist Institute for Biomedical Innovation at Harbor-UCLA Medical Center, Rm 209 Martin Research Building, 1124 West Carson Street, Torrance, CA 90502 USA; 2 Department of Physiology and Pharmacology, Alberta Children’s Hospital Research Institute, Hotchkiss Brain Institute, Cumming School of Medicine, University of Calgary, Rm 203 Heritage Medical Research Building, 3330 Hospital Drive NW, Calgary, Alberta T2N 4N1 Canada

**Keywords:** TRPV1, Lysophosphatidic acid, Asthma, Carotid body, Petrosal ganglion, Allergen, Neuro-immune, Neuroimmunology

## Abstract

The carotid bodies are multimodal sensors that regulate various autonomic reflexes. Recent evidence demonstrates their role in immune reflex regulation. Our previous studies using the allergen (ovalbumin) sensitised and exposed Brown Norway rat model of asthma suggest that carotid bodies mediate asthmatic bronchoconstriction through a lysophosphatidic acid (LPA) receptor (LPAr)-protein kinase C epsilon (PKCε)-transient receptor potential vanilloid one channel (TRPV1) pathway. Whilst naïve carotid bodies respond to LPA, whether their response to LPA is enhanced in asthma is unknown. Here, we show that asthmatic sensitisation of Brown Norway rats involving repeated aerosolised allergen challenges over 6 days, results in an augmentation of the carotid bodies’ acute sensitivity to LPA. Increased expression of LPAr in the carotid bodies and petrosal ganglia likely contributed to this sensitivity. Importantly, allergen sensitisation of the carotid bodies to LPA did not alter their hypoxic response, nor did hypoxia augment LPA sensitivity acutely. Our data demonstrate the ability of allergens to sensitise the carotid bodies, highlighting the likely role of the carotid bodies and blood-borne inflammatory mediators in asthma.

## Introduction

Multimodal neuronal sensors in the carotid bodies signal a host of autonomic reflexes [1]. Known for their oxygen sensitivity [2], growing evidence demonstrates their immune sensing capability and coordination during bacterial infection [3–6]. However, their role in allergen sensing is only beginning to be unravelled. We have demonstrated the role of carotid bodies in eliciting asthmatic bronchoconstriction [7]. We showed that the carotid bodies were sufficient to produce bronchoconstriction by stimulating efferent parasympathetic activity. This bronchoconstriction was instigated, in part, by increased plasma lysophosphatidic acid (LPA) which activates LPA receptors (LPAr) and transient receptor potential vanilloid one (TRPV1) channels in the carotid body-petrosal ganglion complex [7]. We further showed that this pathway likely involved protein kinase C epsilon (PKCε) which served as an intracellular link between LPAr subtype 1 and TRPV1 [8]. The carotid bodies are sensitised by acute intermittent hypoxia after chronic intermittent hypoxia [9] and by acute intermittent hypoxia-hypercapnia without chronic intermittent hypoxia [10]. Sensitisation by acute intermittent hypoxia-hypercapnia without chronic intermittent hypoxia is dependent on TRPV1 activation. Moreover, carotid body hypoxic sensitivity is enhanced by inflammation associated with chronic heart failure [11] and chronic obstructive pulmonary disease [12]. Therefore, we hypothesised that asthmatic sensitisation causing the systemic release of inflammatory mediators acutely sensitises the carotid bodies to phospholipids and hypoxia.

Here, we show that asthmatic carotid bodies have increased LPAr cDNA expression, which appears to increase carotid body activity in response to moderate and high concentrations of LPA. Notably, the hypoxic sensitivity of carotid bodies was similar between naïve Brown Norway (BN) and asthmatic BN rats. We also found similar hypoxic responses between naïve BN and Sprague Dawley (SD) rats, which contrasts with previous reports describing strain differences [9].

## Methods

### Animals and ethical approval

Male BN (p28-35) and SD (p35) rats (Charles River) were housed in a temperature (23 °C) and humidity (30-50%) controlled room with 12/12 light-dark cycle. Food and water were provided ad libitum. Experimental procedures were approved by the University of Calgary Animal Care and Use Committee, in accordance with the Canadian Council of Animal Care, protocol AC15-0061 and AC16-0204.

### Asthmatic model

**Fig. 1:**
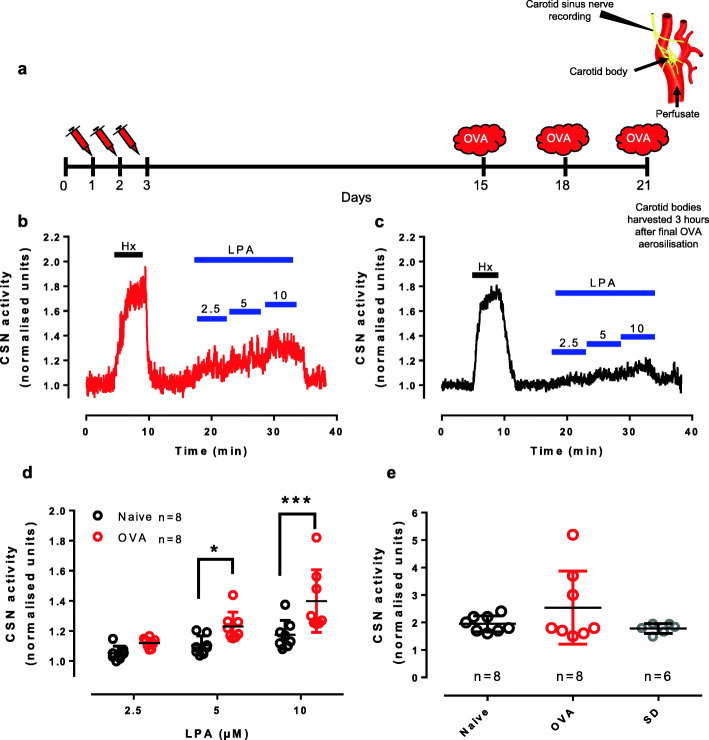
Asthmatic BN rats have increased carotid body sensitivity to LPA. **a** The ovalbumin sensitisation protocol where Brown Norway rats were sensitised (days 1-3) and exposed to aerosolised allergen (days 15, 18, 21) and experiments run 3 h after the last aerosolisation. The en bloc perfused carotid body preparation used to record chemosensory afferents in the carotid sinus nerve (CSN, **a**). OVA rats (**b**) had increased sensitivity to 5 and 10 μM LPA compared to naïve BN rats (**c**). **d** The increase in CSN activity in response to doubling doses of LPA, (2.5, 5, 10 μM, 18:1 LPA) in ovalbumin sensitised and challenged (OVA) and naïve Brown Norway (BN) rats. F_1,14_ (group) = 10.077, *p* = 0.007; F_2,28_ (dose) = 28.430, *p* < 0.001; F_3,63_ (groups × LPA dose) =4.469, *p* = 0.021; post hoc (difference between groups): 2.5 μM, *p* = 0.227; 5 μM, *p* = 0.025; 10 μM, *p* < 0.001. Hx = hypoxia test of viability. **e** The hypoxic response was similar between OVA (*n* = 8) and naïve (*n* = 8) BN rats. When comparing to hypoxic responses from carotid bodies harvested from Sprague Dawley rats (*n* = 6), no difference was difference between rat strain or health status. F_2,19_ = 1.672, *p* = 0.214

BN rats were sensitised to ovalbumin (0.1 mg, Sigma) with pertussis toxin (0.5 ng, Sigma) and aluminium hydroxide as adjuvant (0.15 g, Sigma) dissolved in saline (1 mL); days 1, 2 and 3 (*ip*) and challenged with 5% aerosolised ovalbumin (Fig. 1a), giving an asthmatic phenotype resembling human asthma [13, 14] as established [7, 8]. Naïve BN rats were treated with saline.

### En bloc perfused carotid body preparation

Three hours after final aerosolisation, rats were placed in a bell jar with gauze soaked in isoflurane. Once deeply anesthetised, each animal was euthanised by decapitation and exsanguination; then, the carotid body and carotid sinus nerve were quickly removed, mounted onto a custom stage, perfused and prepared for extracellular recording as established [7, 8, 15, 16]. All preparations were exposed to a brief hypoxic challenge (60 Torr O_2_); preparations that failed to increase nerve activity by 50% during this challenge were discarded. Upon stabilisation, carotid bodies were exposed to hypoxia, then doubling doses of 18:1 LPA (2.5, 5, 10 μM, Cayman Biochemical). In SD rats, responses to LPA (5μM) during normoxia and hypoxia were examined.

### qPCR

Carotid bodies, petrosal ganglia, superior cervical ganglia and nodose ganglia were taken from asthmatic and naïve BN rats (*n* = 6 ganglia of each type were pooled to increase RNA yield) 3 h after their last aerosol challenge. Total RNA (200 ng confirmed with NanoDrop; Thermo Scientific) was converted to cDNA. PCR amplification was carried out in triplicate (20 μl—1μl mRNA, 7 μl ddH_2_O, 1 μL primer/each, and 10 μL Powerup SYBR; ThermoScientific) 50 °C/2 min, 95 °C/2 min followed by 45 cycles of 95 °C/3 s, 60 °C/30 s and melt curve (0.1 °C/s; 60-95 °C; QuantStudio 3, Applied Biosystems). Custom primer sequences are listed in Table 1 (Integrated DNA Technologies). Comparisons were made using 2^∆ctt^ with hypoxyribosyltransferase (HPRT) as housekeeping gene [17].

**Table 1:**
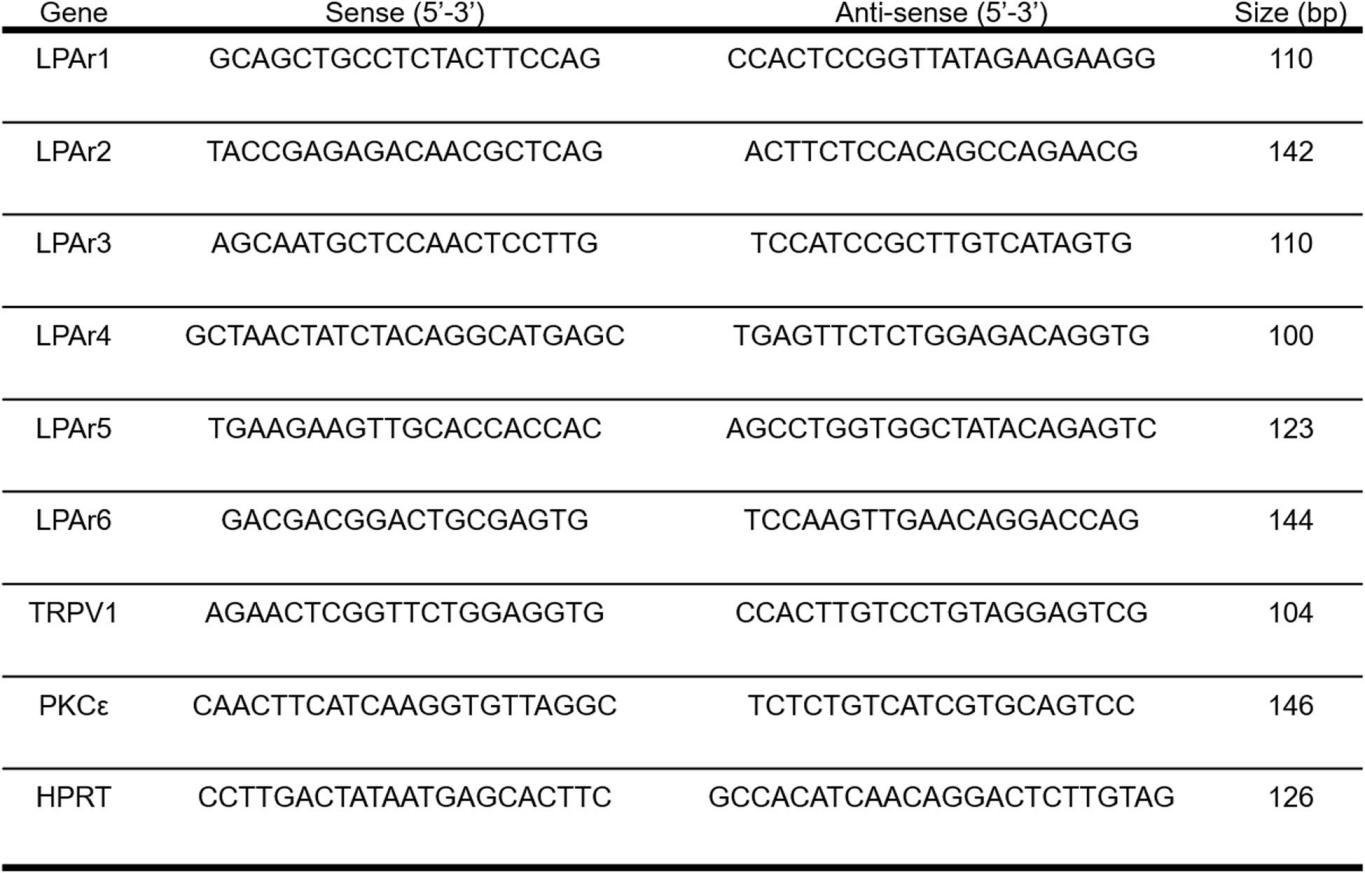
Primer sequences (rat tissue)

### Immunohistochemistry

Carotid bodies were taken from asthmatic and naïve BN rats (*n* = 3 each) 3 h after their last aerosol challenge and fixed in 4% paraformaldehyde, cryoprotected in 30% sucrose and embedded in OCT. Sixteen-micrometre sections were stained with antibodies for LPAr1 (Sigma SAB4500689 1:200), tyrosine hydroxylase (standard glomus cell marker—Sigma ZMS1033 1:200) and appropriate secondary antibodies (Alexa 488 Abcam150116 and Alexa 594 Abcam150077 both 1:200) with DAPI (Sigma MBD0015). Images were visualised using the Leica Thunder 3D tissue culture imaging system.

### Statistics and analysis

Normally distributed data were analysed using parametric two-sided two-way ANOVA, one-way ANOVA with Student-Newman-Keuls post hoc test corrected for multiple comparisons or *T* test as appropriate. Data are presented as mean ± sd; *p* value < 0.05 was considered statistically significant.

## Results

**Fig. 2:**
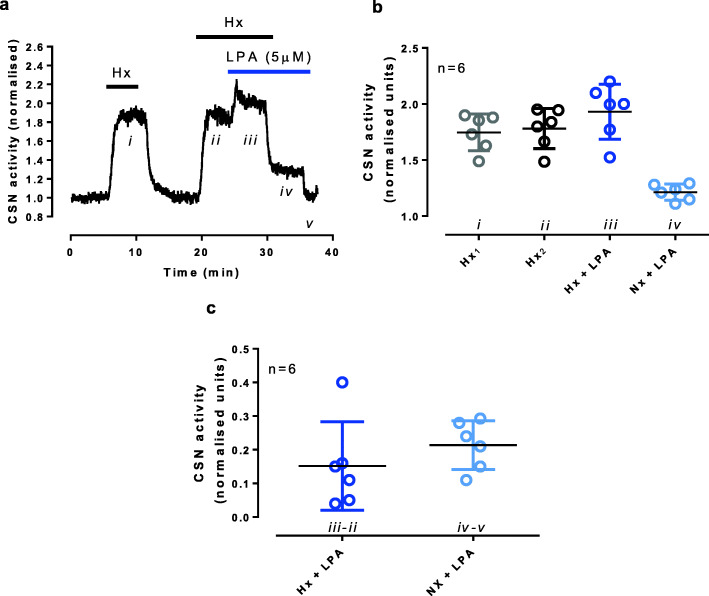
Lysophosphatidic acid stimulation is independent of hypoxic carotid body excitation. **a** A representative trace showing two hypoxic bouts (60 Torr, Hx) where LPA (5 μM) was given during the second hypoxic response, and normoxia (Nx) was re-established with LPA. **b** Summary data illustrating steady state responses during each stimulation. (**c**) The change in carotid sinus activity due to LPA was not different between hypoxia (*iii—ii* from (**a**)) and normoxia (*iv—v* from (**a**), *n* = 6), *t*_10_ = 1.015, *p* = 0.334

Previously, we demonstrated that carotid bodies respond vigorously to endogenous LPA concentrations in a dose- and species-dependent manner [7]. Here, we show that asthmatic carotid bodies have a higher sensitivity to LPA at 5 and 10 μM than naïve carotid bodies (Fig. 1b-d). Hypoxic sensitivity was similar between sensitised asthmatic OVA BN and non-sensitised naïve BN rats and normal SD rats (Fig. 1e). Hypoxia did not augment the LPA (5 μM) response (Fig. 2a-c).

**Fig. 3:**
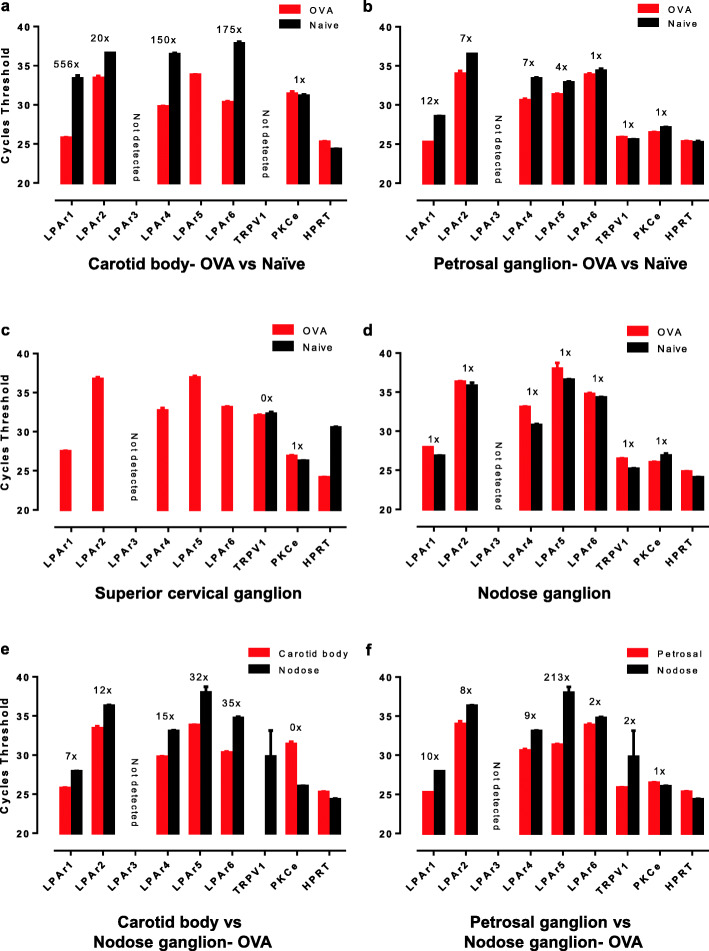
Lysophosphatidic acid receptors are upregulated in asthmatic rats. qPCR reveals that lysophosphatidic acid receptor (LPAr) 1, 2, 4, 5, 6 mRNA was upregulated in the carotid bodies (**a**), and petrosal ganglia (**b**) of ovalbumin sensitised and challenged (OVA) rats compared to naïve Brown Norway (BN) rats. TRPV1 was not detected in the carotid body (**a**) as demonstrated previously [15] and was not increased in OVA compared to naïve BN rats in the petrosal ganglion (**b**). PKCε was detected in all tissues analysed but was not increased in OVA compared to naïve BN rats (**a-b**). **c** LPAr 1, 2, 4, 5, 6 were only detected after 45 cycles in OVA but not naïve BN rats in the superior cervical ganglion. **d** No discernible increases in LPAr expression were revealed in the nodose ganglion. TRPV1 was detected in the superior cervical ganglion (**c**) and nodose ganglion (**d**) but was not increased in OVA compared to naïve BN rats. In OVA rats, the carotid body (**e**) and petrosal ganglion (**f**) demonstrated increased LPAr and TRPV1 mRNA expression in comparison to the nodose ganglion. Differences in expression were analysed by the 2^∆ctt^ method [17] in reference to hypoxyribosyltransferase (HPRT) housekeeping gene. The 2^∆ctt^ number indicating an increase in of red verses black bars are expressed above bars for specific receptor

In SD rats, mRNA for LPAr’s 1, 3, 4 and 6, but not TRPV1, are expressed within the carotid body and mRNA for LPAr’s 3, and 6, and TRPV1 are expressed in the petrosal ganglia (containing the somas of carotid body afferents) [7]. LPAr’s 1, 2, 4 and 6 mRNA are expressed within the carotid body of naïve BN rats, and the mRNA of all four receptors are upregulated in sensitised OVA BN rats (Fig. 3a). Asthma also induced detectable LPAr5 mRNA levels within the carotid body (Fig. 3a). No TRPV1 mRNA was detected within the carotid bodies of naïve or asthmatic rats (Fig. 3a), congruent with our previous findings [15]. LPAr 1, 2, 4, 5, 6 and TRPV1 mRNA are expressed in the petrosal ganglia of naïve animals; asthma causes upregulation of carotid body and petrosal LPAr mRNA (1>4>2>5>6), but the level of PKCε and TRPV1 mRNA expression is unchanged (Fig. 3b).

**Fig. 4:**
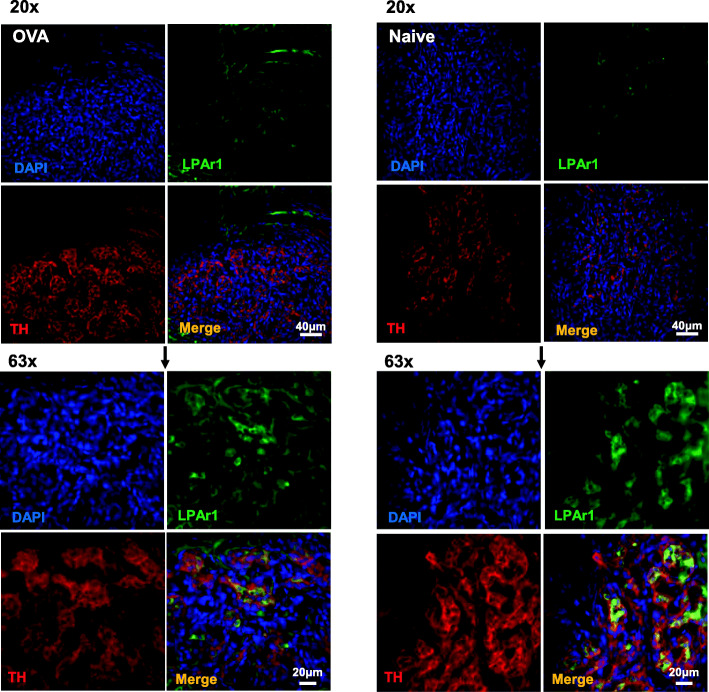
LPAr1 expression in carotid bodies of asthmatic and naïve rats. Lysophosphatidic acid receptor subtype 1 (LPAr1) is predominantly expressed in tyrosine hydroxylase (TH) positive cells which represent glomus cells of the carotid bodies (round cells with large nuclei as imaged with DAPI nuclear stain). Carotid sinus nerves also stain positive for TH and are seen as thin neural axons. More prominent LPAr1 staining in asthmatic OVA BN rats compared to naïve BN rats is consistent with qPCR data from Fig. 3. Top images = 20× magnification, bottom images = 63× magnification

No significant differences in LPAr mRNA expression were observed in the nodose ganglion between asthmatic and naïve BN rats (Fig. 3d). Consequently, asthma-induced increased expression of LPAr mRNA within the carotid bodies and petrosal ganglion compared to the nodose ganglion of asthmatic BN rats (Figs. 3e and f). LPAr1 was predominantly localised to tyrosine hydroxylase (TH-glomus cell marker) expressing cells within the carotid bodies with some staining on thin neuronal projections which appears to be consistent with our qPCR demonstration of LPAr in both the carotid body and petrosal ganglion (Fig. 4).

## Discussion

We previously demonstrated an important role of the carotid bodies in regulating asthmatic bronchoconstriction, involving an LPAr-PKCε-TRPV1 pathway [7, 8]. Here, we show that repeated allergen challenges sensitise the carotid bodies to LPA, likely due to increased LPAr expression in the carotid bodies and petrosal ganglion. Importantly, asthmatic carotid body hypoxic sensitivity is similar to naïve BN and SD rats.

LPA concentrations reflective of asthmatic heightened arterial plasma levels augments carotid body excitability, and arterial plasma harvested from asthmatic rats, presumably rich in LPA, stimulates naïve carotid bodies via an LPAr-PKCε-TRPV1 pathway [7, 8]. The present study suggests that this pathway is sensitised, partly because of distinct increases in LPAr expression in both the carotid bodies and their post-ganglionic site, the petrosal ganglia. LPAr expression in naïve rats was scant as it appeared at ~35 cycles but was increased in asthmatic carotid bodies and petrosal ganglia as LPAr appeared at < 25 cycles. In contrast, PKCε remains relatively unchanged (~25 cycles) in asthmatic and naïve BN rats in the carotid bodies, petrosal, superior cervical and nodose ganglia in relation to the housekeeping gene (~25 cycles). The maintenance of TRPV1 expression was also similar between asthmatic and naïve rats in the petrosal, superior cervical and nodose ganglia in relation to the housekeeping gene. This demonstrates that the terminal portions of this pathway are in relative abundance, but the initial sensing of ligands is adjusted with asthmatic sensitisation. These data are consistent with the localization of LPAr1 to glomus cells with sparse staining on what are likely neurons from the carotid sinus nerve projecting to the petrosal ganglion in both asthmatic OVA and naïve BN rats. Notwithstanding our findings that PKCε and TRPV1 expression are unchanged in all tissue analysed, we cannot exclude the possibility that PKCε and TRPV1 are sensitised by alternate mechanisms such as phosphorylation or a change in cellular location which may contribute to a more predominant role of TRPV1 in the nodose ganglion. Alternatively, others have suggested that TRPA1 regulates vagal (nodose) mediated bronchoconstriction [18]; debate regarding the prominence of TRPV1 and TRPA1 in the nodose ganglion-mediating bronchoconstriction has not been settled [19].

We also tested the expression of the LPAr-PKCε-TRPV1 pathway in the nodose ganglia, which contain cell bodies of lung sensory neurons that are likely to be critical for vagal-mediated asthmatic cough. Unlike the carotid body of asthmatic BN rats, TRPV1 is upregulated in asthmatic guinea pig nodose ganglia [20]. The difference in modulation of these pathways may reflect their different mechanism of activations or roles; the carotid body pathway is activated by blood-borne inflammatory mediators rather than local inflammatory signals in the lung.

We previously demonstrated that following asthmatic provocation, arterial LPA concentrations rise above 5 μM [7]. In this study, we have shown that 5 μM LPA is the threshold by which carotid bodies from asthmatic and naïve animals respond differently. This is congruent with the idea that increases in LPA in the blood caused by allergen challenge of sensitised lungs activate the carotid body-vagal efferent bronchoconstriction pathway. Alternatively, hypoxia may have altered baseline membrane potential and increased the LPA response. However, this possibility is unlikely as hypoxia did not increase the acute LPA response in SD rats.

Hypoxia is a bronchoconstrictor and augments bronchoconstriction during inhaled provocation [21–24], which is counteracted with concurrent hypercapnia [25, 26], a potent bronchodilator [27, 28]. Differences in carotid body oxygen sensitivity may also drive asthmatic bronchoconstriction in conjunction with blood-borne inflammatory mediators. Here, we show no difference between asthmatic and naïve or SD rat carotid body hypoxic sensitivity, in contrast to previous reports of species differences in carotid body hypoxic sensitivity [9]. We suggest this is likely due to ex-vivo carotid body preparation differences between perfused (current study) and superfused (previous study) preparations. Perfusion allows stimulus delivery through the capillaries whereas superfusion relies on the diffusion of stimuli from the periphery of the preparation through connective tissue. Whilst technically easier, superfused preparations have unstirred layers at their boundary that will differ from preparation to preparation. Moreover, the reliance of superfused preparations on diffusion dictates a tissue PO_2_ gradient across the tissue. This suggests that blood-borne inflammatory mediators are a prime stimulus for carotid body-mediated bronchoconstriction, as recently suggested [8].

In summary, we demonstrate that repeated asthmatic allergen exposure in vivo results in upregulation of LPAr and sensitisation of the carotid bodies to LPA, a key ligand responsible for stimulation of carotid bodies in asthma to induce bronchoconstriction [7, 8]. These data highlight the carotid bodies’ role in asthma and support their involvement in allergic immune sensing.

## Data Availability

The datasets used and/or analysed during the current study are available from the corresponding authors on reasonable request.

## Abbreviations

BN: Brown Norway rat
cDNA: Complementary DNA
LPA: Lysophosphatidic acid
LPAr: Lysophosphatidic acid receptor
mRNA: Messenger RNA
PCR: Polymerase chain reaction
PKCε: Protein kinase c epsilon
SD: Sprague Dawley rat
TRPV1: Transient receptor potential vanilloid subtype 1 channel
